# Hippocampal proteomic changes of susceptibility and resilience to depression or anxiety in a rat model of chronic mild stress

**DOI:** 10.1038/s41398-019-0605-4

**Published:** 2019-10-17

**Authors:** Min Tang, Haojun Huang, Shuiming Li, Mi Zhou, Zhao Liu, Rongzhong Huang, Wei Liao, Peng Xie, Jian Zhou

**Affiliations:** 1 Institute of Neuroscience and the Collaborative Innovation Center for Brain Science, Chongqing Medical University, Chongqing, 400016 China; 2 Chongqing Key Laboratory of Neurobiology, Chongqing, 400016 China; 3 Shenzhen Key Laboratory of Microbiology and Gene Engineering, Shenzhen University, Shenzhen, 518060 China; 4 ChuangXu Institute of Life Science, Chongqing, 400016 China

**Keywords:** Depression, Molecular neuroscience

## Abstract

Chronic stressful occurrences are documented as a vital cause of both depression and anxiety disorders. However, the stress-induced molecular mechanisms underlying the common and distinct pathophysiology of these disorders remains largely unclear. We utilized a chronic mild stress (CMS) rat model to differentiate and subgroup depression-susceptible, anxiety-susceptible, and insusceptible rats. The hippocampus was analyzed for differential proteomes by combining mass spectrometry and the isobaric tags for relative and absolute quantitation (iTRAQ) labeling technique. Out of 2593 quantified proteins, 367 were aberrantly expressed. These hippocampal protein candidates might be associated with susceptibility to stress-induced depression or anxiety and stress resilience. They provide the potential protein systems involved in various metabolic pathways as novel investigative protein targets. Further, independent immunoblot analysis identified changes in Por, Idh2 and Esd; Glo1, G6pdx, Aldh2, and Dld; Dlat, Ogdhl, Anxal, Tpp2, and Sdha that were specifically associated to depression-susceptible, anxiety-susceptible, or insusceptible groups respectively, suggesting that identical CMS differently impacted the mitochondrial and metabolic processes in the hippocampus. Collectively, the observed alterations to protein abundance profiles of the hippocampus provided significant and novel insights into the stress regulation mechanism in a CMS rat model. This might serve as the molecular basis for further studies that would contributed to a better understanding of the similarities and differences in pathophysiologic mechanisms underlying stress-induced depression or anxiety, and stress resiliency.

## Introduction

Anxiety and depression are two major debilitating and prevalent psychiatric disorders affecting people of all ages^1–3^. Available research indicates that these two disorders share several putative risk factors, such as severe or stressful events in life and chronic stress^4–6^. Chronic stressful life events are among the environmental factors with a substantial adverse impact on the etiology of depression and anxiety disorders^7,8^. Extreme or sustained exposure to stress often perturbs the individual’s physiological and psychological functioning, leading to a variety of mood- and psychiatry-related disorders mainly including depression and anxiety^7,8^. To model some of the environmental factors affecting humans, the chronic mild stress (CMS) paradigm was developed to induce depression- and anxiety-related behaviors in rodents^7,9,10^. During exposure to CMS, some individuals engage in passive coping behaviors that promote vulnerability to depression and anxiety. Other individuals actively cope with deleterious effects of stress and display resiliency to these disorders^9,10^. The neural substrates underlying vulnerability or resilience to CMS-induced disorders are believed to be meaningful in elucidating the potential biological etiology and pathophysiology of these disorders^11^.

Clinically, depression and anxiety disorders exhibit different core symptoms but frequently coexist^12,13^. In view of considerable comorbidity and pathophysiological overlap of depression and anxiety, recent studies have begun to separately examine non-comorbid subjects with these two disorders in order to unravel common and specific features in the neural systems^14–17^. Depression and anxiety are often marked by memory loss and cognitive impairment, suggesting hippocampal neuronal dysfunction in the pathophysiology of these two disorders^18^. The hippocampus is among the most highly sensitive and malleable brain regions to stress stimulation^19^. Brain imaging data of the hippocampus in patients and stress-induced animal models with either depression or anxiety disorders indicated a remarkable reduction in region volume and dendritic spine numbers^20,21^. Potentially underlying these structural anomalies, chronic stress has been shown to have detrimental effects on hippocampal neurogenesis and neuroplasticity in these individuals^6,22^, consequently leading to cognitive and emotional symptoms of depression and anxiety^23^. Though hippocampal morphological and functional plasticity in both the depressed and the anxious individuals is aberrant^24^, the inherent content and extent of the changes may be substantially different and so far remain obscure. Correspondingly, the molecular regulatory processes linking to the development and maintenance of these disorders remain largely unclear^25^. Thus, there is a pressing need to identify common and distinct molecular characteristics that underlie susceptibility and resilience to depression or anxiety^14^.

It’s well known that proteins are the main executors of physiological function in organisms^26^, and thus their quantitative data/analysis can help enhance our understanding of pathophysiological mechanisms. Proteomics, as an unbiased quantitative tool of protein expression, has been frequently applied to identify a series of significant pathophysiological changes^27^. Moreover, proteomics aids in detecting novel signaling networks that are not easily identified by direct biochemical methods for target identification. We implemented an isobaric tags for relative and absolute quantitation (iTRAQ) based quantitative proteomics approach to explore common and distinct hippocampal protein features associated with depression and anxiety phenotypes in the CMS rat model. This model demarcates depression-susceptible, anxiety-susceptible, and insusceptible subpopulations, representing the three different responses to CMS. Subsequent proteomics analysis revealed several protein changes correlated with characteristics of maladaptive behavior of depression or anxiety disorders as well as adaptive behavior. Our findings provide a critical knowledge base of the common and unique molecular mechanisms underlying stress-induced depression and anxiety, and stress resiliency.

## Materials and methods

### Animal subjects

The Animal Facility of Chongqing Medical University, China provided male Sprague–Dawley rats weighing about 250 g each. They were exposed to a 12 h light/dark cycle at 55 ± 5% humidity and 21–22 °C temperatures with a standard rodent diet and tap water available ad libitum. The National Institutes of Health Guidelines for the Care and Use of Laboratory Animals were followed and the Ethics Committee of Chongqing Medical University approved this study’s protocol. Moreover, all rats were monitored on a daily basis.

### CMS procedure

All rats were single-housed and permitted one week to acclimatize. For sucrose habituation, they were provided water and 1% sucrose solution for two weeks. According to the final three baseline tests of sucrose intakes, the rats were randomly divided into stressed and unstressed groups. The stressed group was exposed to an 8-week CMS procedure as described in Supplementary Table S1^28^. Stressors were applied to induce depression-like and anxiety-like behaviors and included the following: paired housing, a 45° cage tilt, a soiled cage, white noise, water deprivation, an empty water bottle, strobe light, and continuous lighting. In contrast with water deprivation, or the absence of a water bottle, animals given their empty water bottles retain the behavior of water intake.

### Sucrose preference test (SPT) and body weight

The SPT was employed to assess depression-like behavior (anhedonia) of rats. SPT was conducted according to the method described previously^10^. Briefly, during a no-stress period of 24 h, all rats had access to a choice of drinking bottles, one with water and one with 1% sucrose solution. The location of two drinking bottles was alternated arbitrarily between the left and right sides of each cage. The two bottles were weighed before and after testing to measure fluid consumption. Preference for sucrose was calculated using the formula: % sucrose preference = (sucrose intake/total fluid intake) × 100. Prior to the CMS procedure, the body weights of rats were recorded and taken weekly during the CMS period.

### Forced swimming test (FST)

The depression-like behavior (behavioral despair) of rats was assessed with a FST. The test was performed over two sessions as described previously^10^. The rats were placed individually into Plexiglas cylinders of 40 cm in height, and 20 cm in diameter, that were filled with water (temperature 24 °C, height 30 cm), and given a pre-exposure to swimming for 5-min to confirm rapid adoption of an immobile posture. After 24 h of the rats were re-placed into the cylinder for 5 min. A trained observer blinded to group recorded the total immobility time. This test was real-time monitored with a video surveillance system.

### Elevated plus maze test (EMT)

EMT is used to assess anxiety-like behavior of rats, as described previously^7,29^. The elevated plus maze room comprised of two open/close arms designed in a cross-shape. An open central square area of 5 × 5 cm in the arms joined to enable the rats to easily enter both arms of the maze. The rats freely explored the maze for 5 min after placing them onto the open square area, facing an open arm of the maize. We measured the time each rat spent in the open arms, the number of times they entered the open arms and the distance they traveled in each arm.

### Tissue collection and protein extraction

Post behavior test, the rats were decapitated and their brain was immediately removed. The hippocampus tissues were extracted bilaterally and quickly frozen in liquid nitrogen and stored at −80 °C. For protein extraction, the hippocampus tissues were homogenized in SDT buffer (150 mM Tris–HCl, pH 8.0, 4% SDS, 100 mM dithiothreitol, containing a protease inhibitor cocktail). These extracts were boiled for 5 min and centrifuged for 15 min at 40,000 × *g*. The protein concentrations in the supernatant were measured by bicinchoninic acid assay (Pierce, USA).

### Protein digestion

Sixty micrograms of protein lysates from each sample were separated via short gel electrophoresis. As described previously^28,30^, electrophoresis was conducted on a vertical polyacrylamide slab gel consisting of a staking layer of 4%, a separation layer of 10% about 1-cm-long, and a 50% interception layer. A 3.3% of N, N’-methylene bis-acrylamide was used as a cross-linker. Post electrophoresis and staining, each lane was excised and further sheared into four small portions and placed in a tube. In preparation for mass spectrometry (MS), gel digestion of the protein was carried out as previously described^28,30^. The gel slices were de-stained with 50% acetonitrile (ACN) in 50 mM NH_4_HCO_3_. After protein reduction and alkylation, the gels were washed several times in H_2_O and 50% ACN. The gel slices were dehydrated with 100% ACN and dried in a Speed Vac. They were re-hydrated with 200 mM triethylammonium bicarbonate containing 0.01 µg/µL trypsin (Promega, USA) and then underwent overnight incubation at 37 °C. The tryptic peptides were extracted using sonication in 67% ACN containing 2% formic acid. Subsequently the collected samples were dried for iTRAQ labeling.

### Eight-plex iTRAQ labeling and strong cation exchange (SCX) fractionation

Tryptic peptides from the in-gel digestion were labeled with iTRAQ eight-plex reagents as previously described^10,28^. Eight samples, each obtained from two rats for the control, depression-susceptible, anxiety-susceptible, and insusceptible groups were labeled with reagents 113–121. They were pooled and then fractionated by offline SCX chromatography strategies, where the peptides underwent fractionation via gradient elution with SCX buffer (25% ACN, 10 mM KH_2_PO_4_, 500 mM KCl, pH 3.0), at 1-ml/min flow rate. The obtained fractions were pooled into 10 groups, desalted, vacuum-dried, and reconstituted with 0.1% formic acid in preparation for MS analysis.

### Liquid chromatography-tandem mass spectrometry (LC-MS/MS)

The peptides in the fractions were detected by use of AB SCIEX TripleTOF 5600 + mass spectrometer coupled with a splitless NanoLC-Ultra 2D Plus system (Eksigent, Dublin, CA, USA)^28,30^. This Nanoflex system uses a C18 desalting column (100 μm × 3 cm, 3 μm) and a ChromXP C18 (3 μm, 120 Å) packed separation column (75 μm × 15 cm). The desalted and loaded peptide solution was segregated using an elution gradient of 70-min at a 300-nl/min flow rate. The elution gradient began with a 5% mobile phase B (98% acetonitrile/0.1% formic acid) which progressed to 35%. Acquisition of MS data was carried out using an information-dependent acquisition (IDA) mode, where a mass range of 350–1,250 Da was scanned via a 250-ms IDA survey. After, 35 MS/MS scans of 100-ms at a 120 cps (counts/s) trigger with a +2 to +5 precursor charge state were carried out. A FWHM resolving power of 30,000 was used to conduct the MS scans, along with an 18 s dynamic exclusion time. Based on the charge-to-mass ratio of precursor ions, the optimal collision was computed empirically during the MS scan and the enhanced iTRAQ function was switched on to increase collision-induced dissociation efficiency.

### Data analysis

AB SCIEX ProteinPilot software 4.5 with Paragon database search algorithm (4.5.0.0.1654) was employed for protein identification and iTRAQ quantitation^28^. The freely-available UniProt rat database released in July 2016 was imported for the search and it was merged with false discovery rate (FDR) analysis. The search parameters used included: sample type (eight-plex iTRAQ), digestion (trypsin), up to 2 missed cleavages, carbamidomethyl cysteine for fixed modification, methionine oxidation for variable modification, a peptide mass tolerance of ±20 ppm, 0.1 Da of fragment mass tolerance, TripleTOF 5600 instrument, and detailed ID for search effort. First, a 95% confidence was used to filter the peptides. Then, the ProGroup algorithm for redundancy minimization was used to group the proteins. Proteins were identified based on an unused protein score cutoff of ≥1.3, an FDR < 1%, two or more unique peptides with at least 95% confidence, or a single peptide with at least 99% confidence (Supplementary Table S2), as previously described^31,32^. Further, the relative quantity of a peptide across the diverse samples was assessed by comparing the intensities of reporter ion signals in the MS/MS scan. The protein ratios were computed from the average of all suitable isotope-tagged peptide ratios. A bias correction for the possible systematic error of unequal mixing in the different labeled samples was carried out based on the assumption that most proteins do not alter in expression. The software took the mean protein ratio and corrected it to unity. This is then applied to all quantification data. The data were exported into Excel for manual interpretation and then the protein ratios underwent a two-tailed Student’s t-test. Proteins with mean ratios of >1.2 and <0.83 and *p*-values of <0.05 were considered as significantly differentially expressed^28^. Following the data-sharing policy of the ProteomeXchange consortium, all raw and metadata have been deposited in the iProx submission system (http://www.iprox.org) and can be accessed with the identifier IPX0001464001.

### Bioinformatics analysis

A multi-omics tool (OmicsBean) (http://www.omicsbean.cn) was employed for in silico analysis of the identified differentially expressed proteins^33^. The enrichment analyses of these proteins in biological process (BP), cellular component (CC), and molecular function (MF) were conducted based on the Gene Ontology (GO) terms of each protein. Further, Kyoto Encyclopedia of Genes and Genomes (KEGG) pathway analysis was employed and the pathways or GO terms with *p*-value <0.05 were considered significant^34^. Additionally, evaluation of protein–protein interactions was conducted with STRING database and visualized using Cytoscape software, as described previously^33^.

### Immunoblot analysis

Immunoblots were conducted on hippocampal protein extracts according to our previously described procedure^28,35^. The primary antibodies used included: Esd (1:10,000; ab133631, Abcam), Idh2 (1:1,000; ab131263, Abcam), Dld (1:10,000; ab133551, Abcam), Sdha (1:1,000; ab137040, Abcam), G6pdx (1:1,000; ab210702, Abcam), Dlat (1:1,000; ab172617, Abcam), Pkm (1:1,000; C103A3, CST), Aldh2 (1:1,000; ab108306, Abcam), Glo1 (1:1,000; ab137098, Abcam), Ogdhl (1:500; 17110–1-AP, Proteintech), Anxal (1:2,000; ab214486, Abcam), Por (1:10,000; ab180597, Abcam), Prdx6 (1:1,000; ab133348, Abcam), Prnp (1:5,000; ab52604, Abcam), and Tpp2 (1:1,000; ab180177, Abcam). Anti-mouse or anti-rabbit horseradish peroxidase-conjugated IgG (1:10,000; Bio-Rad) was used as a secondary antibody. After gel electrophoresis and immunodetection, the protein band intensities were analyzed using Quantity One (Bio-Rad) software. Non-target protein bands in Coomassie blue-stained gels before being transferred to a PVDF membrane were used as the loading control^28,36^.

### Statistical analysis

SPSS software was used for all statistical analysis^10^. One-way analyses of variance (ANOVA) followed by the post hoc least significant difference test was used to analyze the SPT, FST, EMT, body weight, and immunoblot data. *P*-values of <0.05 were considered statistically significant. All results were expressed as mean ± standard error.

## Results

### Behavioral assessment of CMS rats

**Fig. 1:**
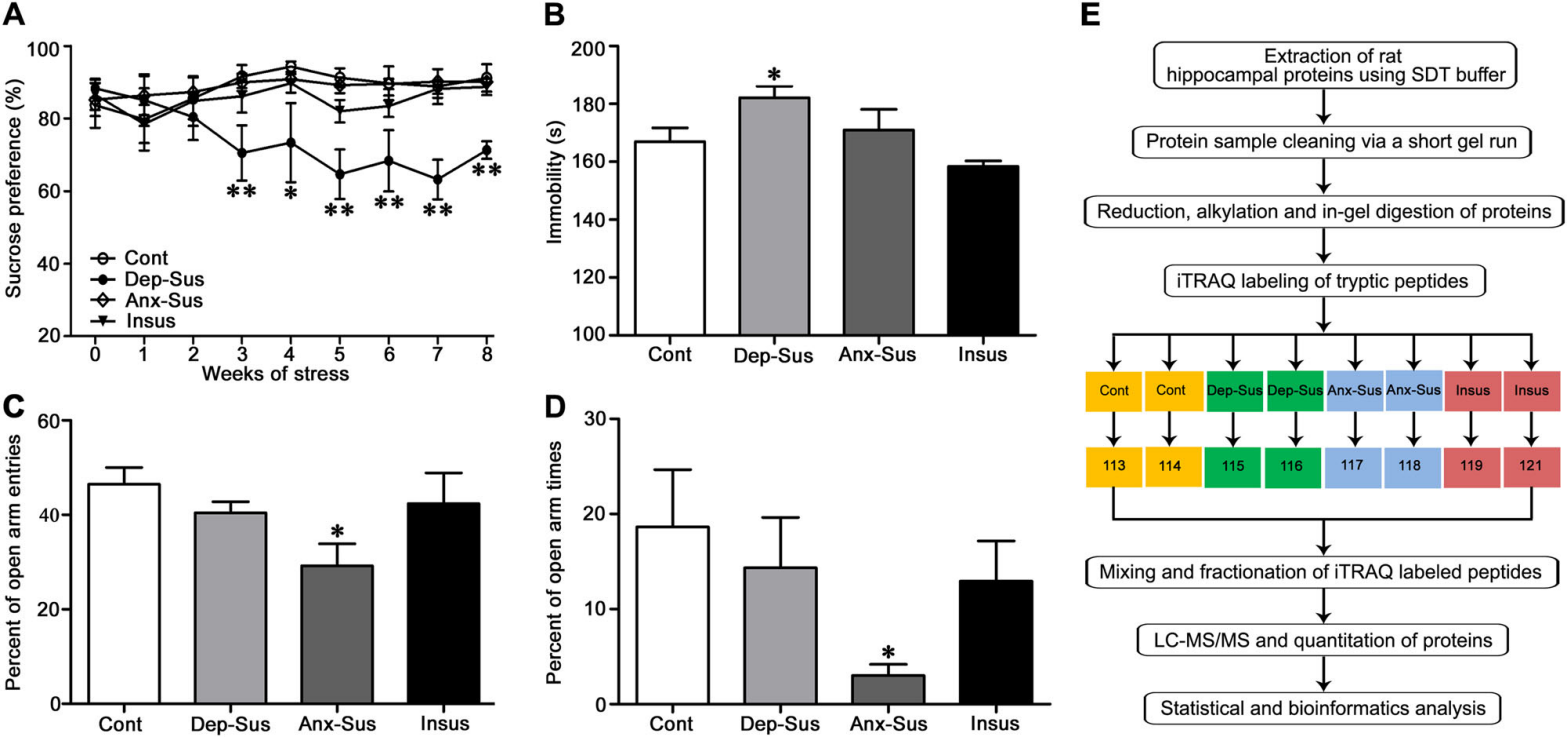
Behavioral assessment of the chronic mild stressed (CMS) rat model and isobaric tags for relative and absolute quantitation (iTRAQ)-based proteomic analysis of hippocampal proteins. The results of the (**a**) sucrose preference test (SPT) and (**b**) forced swim test (FST) for depression-related behavioral assessment and (**c**, **d**) elevated plus maze test (EMT) for anxiety-related behavioral assessment. (**e**) Workflow diagram followed in our study. Proteins were subjected to iTRAQ labeling followed by mass spectrometry (MS) detection. Dep-Sus, Depression-Susceptible; Anx-Sus, Anxiety-Susceptible; Insus, Insusceptible; Cont, Control; *n* = 5, *p < 0.05, **p < 0.01

We first performed the SPT and FST analysis to evaluate the depression-like behavior of CMS-exposed rats, thereby obtaining the depression-susceptible subgroup^33^. Through an eight-week CMS regime, rats displaying a significant reduction in sucrose preference were considered to be susceptible to CMS-induced depression^33^. As shown in Fig. 1a, the depression-susceptible group showed a significantly lower sucrose preference as compared with other groups over the 3-weeks and this difference remained significant through the last week. The FST result indicated higher immobility time for the depression-susceptible group at the end of the CMS procedure compared with the other groups (*p* < 0.01), suggesting that the despair-like behavior of CMS-exposed rats was correlated to anhedonia (Fig. 1b). Of note, some CMS rats did not exhibit depression-like behavior, but rather showed an anxiety-like behavior as indexed by the EMT, and could be considered to be susceptible to stress-induced anxiety^7,37^. It was observed that the anxiety-susceptible group entered fewer times and spent less time in the open arms of the maze when compared to the other groups (Fig. 1c, d). Furthermore, no significant differences in the SPT, FST, and EMT were observed among the control and insusceptible groups, which suggests that the insusceptible group did not have any depression-like and anxiety-like phenotypes. In addition, a gradual but significant increase in body weight in the controls was observed, almost over all weeks, compared with the stressed groups. However, there were no significant differences among the stressed groups (Supplementary Fig. S1). Taken together the behavioral data suggested that a subset of the control, depression-susceptible, and anxiety-susceptible group could be obtained, and the CMS paradigm described here might provide an effective tool for investigating disorder-specific molecular aspects especially the specific and general neural characteristics of non-comorbid depression and anxiety.

### Quantitative proteomic analysis of the rat hippocampal proteins

In this study, changes in the hippocampus proteome of CMS-exposed rats were investigated via employing the previously established iTRAQ-based quantitative approach^10,28^. An outline of the iTRAQ-based quantitative workflow is displayed as Fig. 1e. For comparative proteomics analysis, four animals were used per group and the hippocampus tissues from two rats for each of these samples were pooled as described previously^38^. The whole proteins were effectively extracted using the SDT lysis buffer. Before proceeding with trypsin digestion and MS detection, a short gel-based approach was employed to eliminate SDS interference^28,39^. In the current study, a total of 2593 distinct proteins were identified and 2566 were quantified with less than 1% FDR (Supplementary Table S2). The efficiency of iTRAQ labeling was assessed to be about 97% as described previously. The proteome profiles of control, depression-susceptible, anxiety-susceptible, and insusceptible groups were comparatively analyzed to identify differentially expressed proteins. Proteins with *p* < 0.05 (student’s *t*-test) and a fold change >1.2 were considered significantly expressed. We identified 367 CMS-responsive proteins with these cut-offs (Supplementary Table S2).

### Functional and network analysis of differential protein in CMS-induced rats

**Fig. 2:**
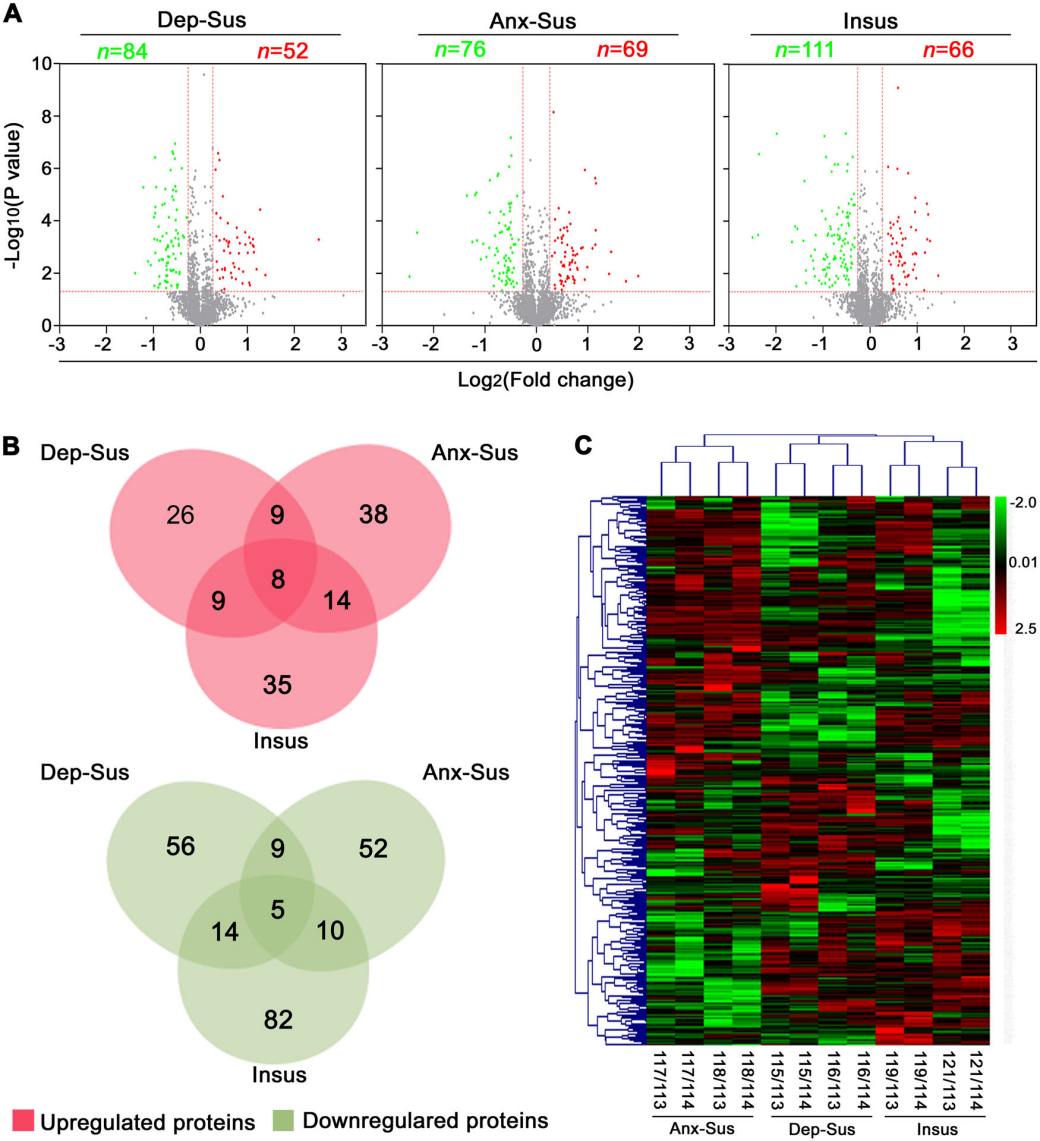
Analysis of the differentially expressed proteins from proteomic profiling. (**a**) Volcano plot showing variation in protein expression in the three groups. The fold change log (base 2) is on the x-axis, and the negative false log discovery rate (*p*-value) (base 10) is on the y-axis. Higher expressions are indicated by red and lower by green. (**b**) Venn diagrams, revealing the number of altered proteins in the three groups. (**c**) Heatmap clustering of differentially expressed proteins in the depression-susceptible, anxiety-susceptible, and insusceptible groups, relative to control. Higher expressions are indicated by red and lower by green. The expression levels are indicated by different intensities of colors, which are represented by the color-key bar with a log2-scale. The x-axis represents the eight available isobaric tags for relative and absolute quantitation (iTRAQ) reagents used. Dep-Sus, Depression-Susceptible; Anx-Sus, Anxiety-Susceptible; Insus, Insusceptible; Cont, Control

Differential proteomic analysis in the rat model revealed 52 up-regulated and 84 down-regulated proteins in the depression-susceptible group, 69 up-regulated and 76 down-regulated proteins in the anxiety-susceptible group, and 66 up-regulated and 111 down-regulated in the insusceptible group. We found 31 proteins with similar regulations in the two susceptible (depression and anxiety) groups, which may represent some common abnormal components between these two CMS-induced disorders. Among the insusceptible and the two susceptible groups, only 13 proteins were regulated similarly as a result of exposure to CMS (Fig. 2b). Interestingly, the association of 79% of these proteins among the three stressed groups, suggested that the molecular mechanisms underlying responses to stress are different among these groups. Further, unsupervised hierarchical clustering analysis of the 367 differentially expressed proteins clustered them into three distinct groups representing the three different responses to CMS (Fig. 2c).

**Fig. 3:**
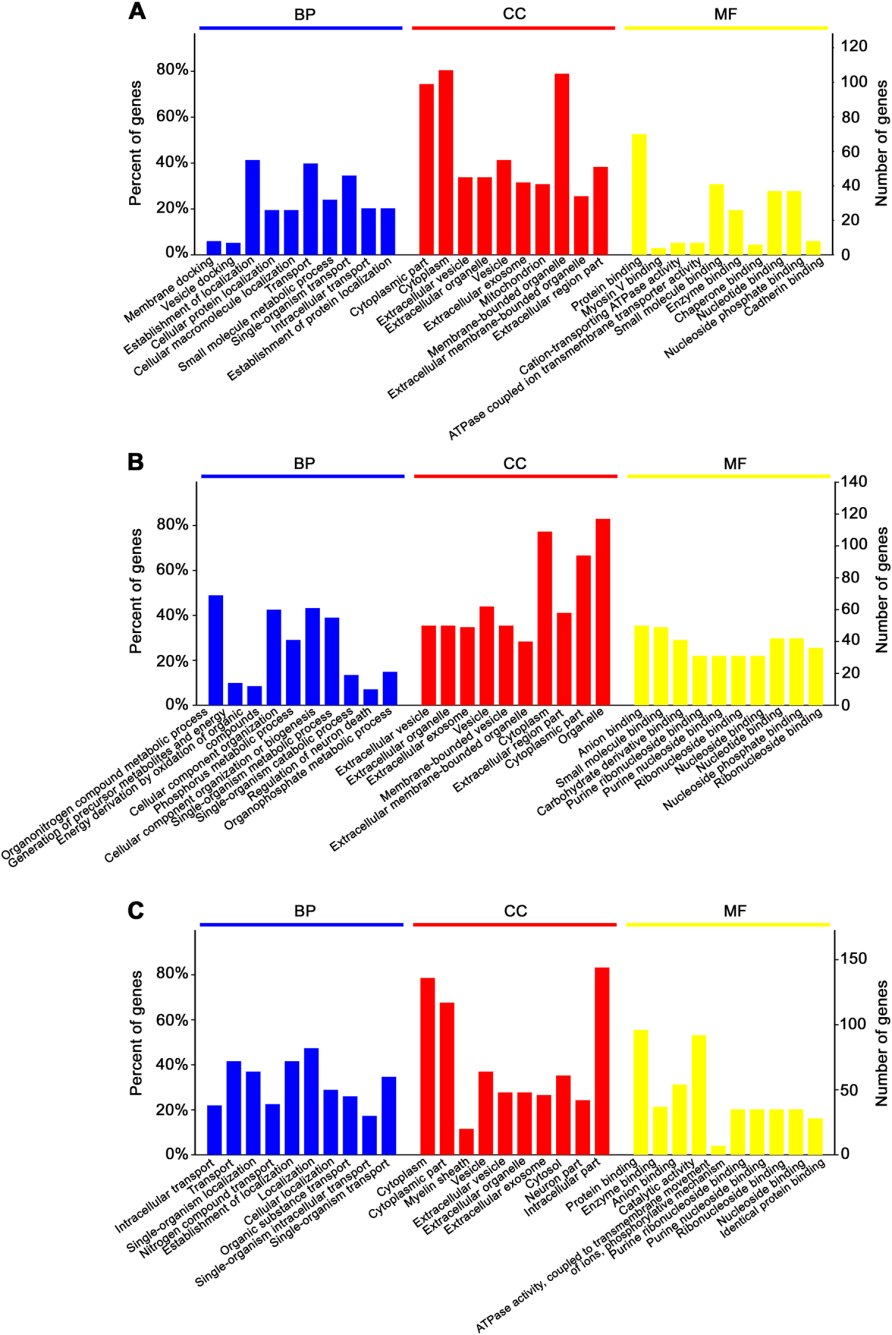
Enrichment analysis of Gene Ontology (GO) pathway terms for the differentially expressed proteins. The top ten enriched terms from the depression-susceptible (**a**), anxiety-susceptible (**b**), and insusceptible (**c**) groups in biological process (BP), cellular component (CC), and molecular function (MF) were identified

**Fig. 4:**
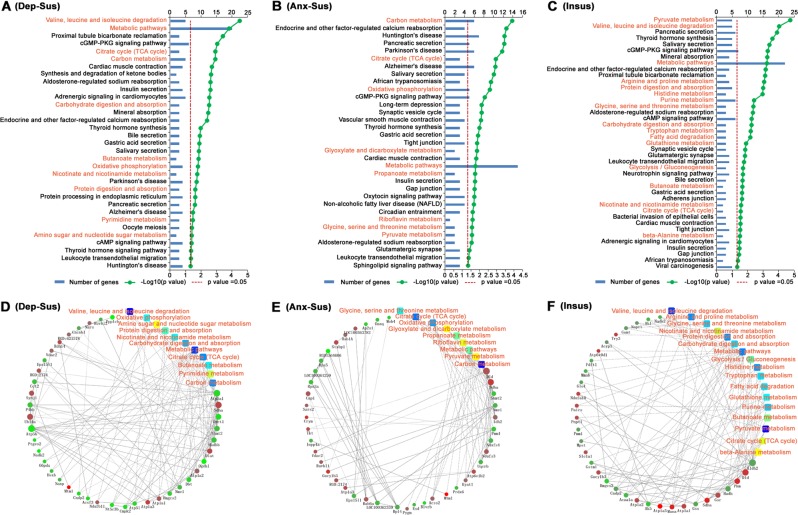
Kyoto Encyclopedia of Genes and Genomes (KEGG) pathway enrichment and protein–protein interaction (PPI) networks of the differentially expressed proteins. The significantly enriched terms from **a** depression-susceptible, **b** anxiety-susceptible, and **c** insusceptible groups were identified and titles in red indicate metabolism-related KEGG pathways. The *p*-value negative log (base 10) is on the *x*-axis. PPI networks from **d** depression-susceptible, **e** anxiety-susceptible, and **f** insusceptible groups. Based on the fold change of a proteins/gene, pathway enrichment by KEGG and enrichment of biological process, the PPI analysis was performed. Circular nodes represent proteins/genes, and the rectangle represents the KEGG pathway or biological process and is colored according to increasing *p*-value (yellow refers to lower *p*-value and blue refers to higher *p*-value)

To gain insights into the involvement of these proteins in different biological functions and pathways, these differentially expressed proteins in each CMS response group were analyzed with the OmicsBean tool^33,34^. GO analysis of the 136 differentially expressed proteins from the depression-susceptible group identified 768, 203, 222, and 33 terms to be significantly enriched (*p* < 0.05) in the biological process (BP), molecular function (MF), cellular component (CC), and KEGG pathway categories respectively (Supplementary Table S3). Figure 3a depicts the top ten enriched terms in the depression-susceptible group. Many proteins in the BP category were associated with docking, transport, and metabolic processes. CC category analysis showed that most of the proteins were predicted to be located in cytoplasm, vesicles, and mitochondria. MF analysis revealed that most of the proteins identified are involved in small molecule, protein, and nucleotide binding and catalytic activity. KEGG pathway enrichment indicated that the identified proteins are mainly involved in valine, leucine and isoleucine degradation, metabolic pathways, proximal tubule bicarbonate reclamation, cGMP-PKG signaling pathway, carbon metabolism, citrate cycle (TCA cycle), etc (Fig. 4a).

The 145 differentially expressed proteins from the anxiety-susceptible group were also categorized according to GO enrichment analysis, and 882, 210, 203, and 33 terms were identified to be significantly enriched (*p* < 0.05) in the BP, CC, MF, and KEGG pathway categories respectively. Figure 3b illustrates the top 10 enriched terms in the anxiety-susceptible group. The majority of proteins in the BP category are involved in the metabolic process of organonitrogen compounds, phosphorus, single-organism, organophosphate, purine ribonucleoside triphosphate, etc. CC category showed protein belonging to vesicle and cytoplasm parts, and MF indicated most protein to be involved in anion, small molecule, carbohydrate derivative, and nucleotide binding. KEGG pathway enrichment revealed these proteins to be linked with carbon metabolism, endocrine, and other factor-regulated calcium reabsorption pathways, Huntington's disease, pancreatic secretion, Parkinson's disease, etc (Fig. 4b).

GO enrichment analysis of the 177 differentially expressed proteins from the insusceptible group identified 1008, 231, 233, and 41 terms to be significantly enriched (*p* < 0.05) in the BP, CC, MF, and KEGG pathway categories respectively. Figure 3c shows the primary 10 enriched terms in the insusceptible group according to GO analysis. The majority of proteins in the BP category are involved in transport and localization. CC category indicated most proteins to be located in the cytoplasm and vesicle parts and MF category showed most proteins to be involved in protein, enzyme, anion, nucleotide binding, and catalytic activity. Pathway enrichment using KEGG revealed that these proteins were linked to pyruvate metabolism, valine, leucine and isoleucine degradation, pancreatic secretion, thyroid hormone synthesis, etc (Fig. 4c).

Interestingly, in the depression-susceptible group, 11 of 33 significantly enriched KEGG pathways were involved in the metabolic processes, which consisted of 7 up-regulated and 18 down-regulated proteins (Fig. 4a). Among the anxiety-susceptible group, 9 of 33 significantly enriched KEGG pathways were associated with metabolism, which consisted of 9 up-regulated and 9 down-regulated proteins. Between these two groups, there were some common and distinct pathways corresponding to significantly dysregulated proteins. Furthermore, we also found that 17 of 41 significantly enriched KEGG pathways in the insusceptible group were involved in the metabolic process, which consisted of 15 up-regulated and 18 down-regulated proteins. This indicated that metabolism-related pathways accounted for a relatively large proportion in the three stressed groups. Of note, despite similarity among the three groups, a significant number of metabolic pathways and corresponding proteins were noted, that were specifically associated to either depression-susceptible, anxiety-susceptible, or insusceptible groups, suggesting differences in the metabolic mechanisms underlying these three phenotypes (Supplementary Fig. S2 and Table S4). Subsequently, we further focused on the proteomics-inferred metabolic networks in the three CMS response groups (Fig. 4d, e). The significantly dysregulated proteins associated with the metabolic process were used in a protein–protein interaction (PPI) network analysis. By means of a unified conceptual framework, 25, 18, and 33 proteins were found to be key nodes in metabolism-related PPI networks from the depression-susceptible, anxiety-susceptible, and insusceptible groups respectively. These networks showed a close association between proteins and metabolic pathways and provided a small pool of metabolism-related interactome in stress susceptibility and resiliency, which may be relevant in future investigations.

### Immunoblot detection of CMS-related mitochondrial and metabolic proteins

**Fig. 5:**
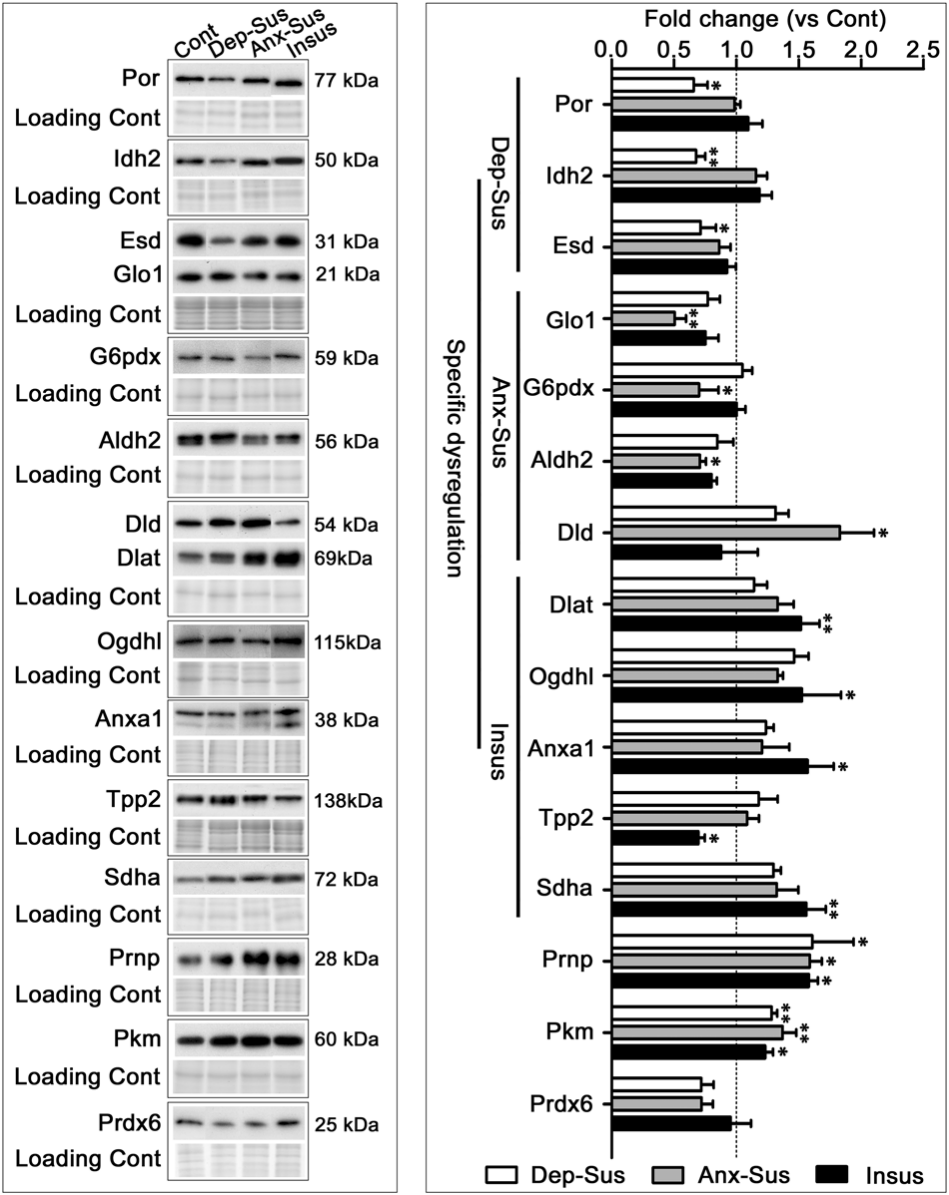
Protein immunoblot analysis. Immunoblotting of the proteins involved in mitochondrial and metabolic functions was performed in the hippocampi from the control, depression-susceptible, anxiety-susceptible, and insusceptible groups. Por, Idh2, Esd, Glo1, G6pdx, Aldh2, Dld, Dlat, Ogdhl, Anxal, Tpp2, Sdha, Prnp, Pkm, and Prdx6 were detected with their respective antibodies. Each blot corresponded to the five rats used in the analysis, and Coomassie blue staining was used as the loading control. *Dep-Sus* depression-Susceptible, *Anx-Sus* Anxiety-susceptible, *Insus* Insusceptible, *Cont* Control; *n* = 5, *p < 0.05, **p < 0.01

Altered mitochondrial and metabolic dysfunctions are generally believed to be important for stress-related disorders^40,41^. In this study, 15 proteins of interest involved in mitochondrial and metabolic pathways were selected for further expression validation by immunoblot to investigate their possible mechanisms. Overall, the immunoblot results mirrored the iTRAQ-based proteomics data (Supplementary Fig. S3). Further statistical analysis demonstrated that Por, Idh2, and Esd were significantly down-regulated in the depression-susceptible group as compared with the control group (Fig. 5). In the anxiety-susceptible group, Glo1, G6pdx, and Aldh2 were significantly down-regulated, while Dld was up-regulated, when compared to the control group. In the insusceptible group, Tpp2 was significantly down-regulated, while Dlat, Ogdhl, Anxal, and Sdha were up-regulated, when compared to the control group. Furthermore, Prnp and Pkm were significantly up-regulated in all three stressed groups as compared to the control group. For some of the proteins, the proteomics and immunoblot results showed discrepancies as reported in other proteomics studies^42–45^. Besides intrinsic differences between the two methods such as procedural steps and dynamic ranges^42^, another contributing factor might be the additional pooling procedure in the proteomics experiment^46^.

## Discussion

The CMS procedure has been suggested to mimic socio-environmental stresses in inducing depression- and anxiety-related behaviors^7,10,33,47,48^. In our study, we identified three different stress-response phenotypes in the CMS rat model, mainly assessed by means of SPT, FST, and EMT^7,10,33^. Some rats exposed to CMS exhibited anhedonia-like behavior where their sucrose preferences were slower compared with the control. They also displayed a sign of behavioral depression, where the rats spent more time being immobile during the FST. Herein these rats were considered as susceptible to stress-induced depression. Furthermore, despite no deficit in the SPT and FST, some rats were more anxious, as they were less willing to stay or the open arms of the elevated plus maze. These rats could be considered as susceptible to stress-induced anxiety. It is noted that some animals did not exhibit depression- or anxiety-like behaviors and showed resiliency to developing these behaviors despite exposure to CMS. Overall, the CMS paradigm simulated realistic conditions for human depression and anxiety and generated several behavioral changes comparable to those witnessed clinically. This indicated that the molecular alterations identified using this rat model may also take place in human patients with stress-induced depression or anxiety. In our study, classification into depression-susceptible, anxiety-susceptible, and insusceptible cohorts provided a valuable approach in identifying common and distinct molecular bases of vulnerability to stress-induced depression or anxiety and stress resiliency. This CMS model can also provide valuable information for translational research.

We carried out iTRAQ-based quantitative proteomics followed by bioinformatics analysis to unravel the neurobiological underpinnings and identify phenotype-specific protein dysregulations in the hippocampi of the CMS rats exhibiting the three different behaviors. In total, 367 differentially expressed proteins were recognized from the hippocampi of depression-susceptible, anxiety-susceptible, and insusceptible groups as compared to the non-stressed controls. The proteins were that were altered similarly between the depression/anxiety-susceptible and insusceptible groups might serve as general functional components involved in the response to CMS. Those overlapping proteins between the depression-susceptible and anxiety-susceptible groups might underlie the common patterns of these two different disorders. Of note, proteins regulated uniquely in the three groups may underlie the observed behavioral differences. The heat map analysis also suggested that the three stressed groups displayed distinctive protein functional profiles. These results may help other researchers discover proteins and pathways associated with stress-induced depression or anxiety and stress resilience^11^.

Our proteomic data implicate affected pathways associated with stress-induced behaviors of depression, anxiety, and resilience in the hippocampus. KEGG pathway analysis indicated that these differentially expressed proteins were prevailingly involved in metabolic processes, such as the tricarboxylic acid (TCA) cycle, carbon, and pyruvate metabolism, oxidative phosphorylation, as well as other important metabolic processes. The regulatory network analysis revealed that protein-mediated metabolic processes as the common denominator may play a role in dysfunctional responses to stress^41^. These findings provide evidence that these metabolic mechanisms may also represent an important factor in the pathophysiological basis of stress. The 15 differentially expressed proteins involved in the metabolic process and selected for further validation with immunoblot indicated that Por, Idh2, and Esd were distinctly disregulated in the depression-susceptible group. Meanwhile, Glo1, G6pdx, Aldh2, and Dld were distinctly dysregulated in the anxiety-susceptible group. The specificity of the hippocampal molecular response suggests that similar CMS can influence the molecular processes differently, potentially prompting different biological repercussions in the hippocampus and correlative behavioral phenotypes in rats. Furthermore, Dlat, Ogdhl, Anxal, Tpp2, and Sdha were distinctly dysregulated in the insusceptible group, probably suggesting an important way to cope with the stress-induced metabolic dysfunctions in the hippocampus. It is tempting to speculate that these alterations may be involved in a stress protection mechanism in the rat hippocampus^9^.

In our present study, the 15 immune-detected proteins are mainly involved in TCA, carbon, and pyruvate metabolism. Among these proteins, we found that 8 proteins are predicted to be located in the mitochondria. In general, mitochondria are essentially involved in numerous metabolic processes, like metabolism of nucleic acid, protein, fat, and glucose^40^. Several pathogenic processes register mitochondrial dysfunction as the key cause including viral infection, autophagy, cell death, and metabolism^49^. Neuronal differentiation requires higher rates of mitochondrial biogenesis and thus impaired neuroplasticity could be a result of dysfunctional mitochondria in the hippocampus of depressed and anxious rats^50–52^. The mitochondrial and metabolic dysfunctions might be associated with the diminished plasticity of neurons and impaired neurogenesis of the hippocampal is considered to be operative during depression and anxiety, as neurogenesis is a process with enhanced metabolic demands^53^. Though we identified alterations in the expression of proteins involved in metabolic and mitochondrial dysfunctions in the hippocampus of the stressed rats, the detailed mechanisms behind such perturbations requires additional investigations.

## Conclusion

Using iTRAQ-based quantitative proteomics to study alterations in the hippocampus of CMS-exposed rats identified several hippocampal protein candidates that might be associated with susceptibility to stress-induced depression or anxiety and stress resilience, and provided potential protein systems particularly involved in mitochondrial and metabolic pathways as new investigative protein targets. This offers novel insights into the regulatory mechanisms behind stress in CMS-exposed rats which may serve as the molecular basis for future research. This research can contribute to a better understanding of the similarities and differences of pathophysiologic mechanisms underlying stress-induced depression or anxiety and stress resiliency.

## Supplementary information

Supplementary information

Supplementary Table S2

Supplementary Table S3

Supplementary Table S4
