# Supplementary material for: Hippocampal proteomic changes of susceptibility and resilience to depression or anxiety in a rat model of chronic mild stress
Source: Transl Psychiatry. 2019 Oct 17;9:260. doi: 10.1038/s41398-019-0605-4 (PMC6797788; doi:10.1038/s41398-019-0605-4)
Supplement: Supplementary file 1 — Supplementary information [file 41398_2019_605_MOESM1_ESM.docx]

**Supplementary Information**

**Hippocampal proteomic changes of susceptibility and resilience to depression or anxiety in a rat model of chronic mild stress**

Min Tang^1,2^, Haojun Huang^1,2^, Shuiming Li^3^, Mi Zhou^1,2^, Zhao Liu^1,2^, Rongzhong Huang^4^, Wei Liao^1,2^, Peng Xie^1,2*^, Jian Zhou^1,2*^

^1^Institute of Neuroscience and the Collaborative Innovation Center for Brain Science, Chongqing Medical University, Chongqing 400016, China

^2^Chongqing Key Laboratory of Neurobiology, Chongqing 400016, China

^3^Shenzhen Key Laboratory of Microbiology and Gene Engineering, Shenzhen University, Shenzhen 518060, China

^4^ChuangXu Institute of Life Science, Chongqing 400016, China

Min Tang, Haojun Huang and Shuiming Li contributed equally to this work.

*To whom correspondence should be addressed:

Institute of Neuroscience and the Collaborative Innovation Center for Brain Science, Chongqing Medical University, 1 Yixueyuan Road, Yuzhong District, Chongqing 400016, China. Tel: +86-23-68485490, Fax: +86-23-68485111.

E-mail addresses: xiepeng@cqmu.edu.cn (P. Xie), zhoujian@cqmu.edu.cn (J. Zhou).

**Supplementary information**

**Supplementary Figure S1.** Change in body weight of the control and stressed groups during eight weeks; n = 5, *p < 0.05, **p < 0.01.

**Supplementary Figure S2.** Venn diagrams showing the number of main metabolic pathways and the corresponding dys-regulated proteins in the depression-susceptible, anxiety-susceptible, and insusceptible groups. More detailed information is provided in Supplementary Table S4. Dep-Sus, Depression-Susceptible; Anx-Sus, Anxiety-Susceptible; Insus, Insusceptible.

**Supplementary Figure S3.** Comparison between isobaric tags for relative and absolute quantitation (iTRAQ)-based proteomic and immunoblot results in this study.

**Supplementary Table S1.** Chronic mild stress (CMS) schedule in the present study.

**Supplementary Table S2.** List of all identified proteins and differentially-expressed proteins from the depression-susceptible, anxiety-susceptible, and insusceptible groups. The differentially-expressed proteins are highlighted in gray.

**Supplementary Table S3.** A complete list of gene ontology (GO) biological process (BP), cellular component (CC), molecular function (MF) and Kyoto Encyclopedia of Genes and Genomes (KEGG) pathway terms for the differentially-expressed proteins identified in depression-susceptible, anxiety-susceptible and insusceptible groups. The terms with p-value < 0.05 are highlighted in gray.

**Supplementary Table S4**. Metabolism-related differential hippocampal proteins and their correlated Kyoto Encyclopedia of Genes and Genomes (KEGG) pathways. Significantly up- or down-regulated proteins in depression- susceptible, anxiety-susceptible, and insusceptible groups as compared to the control are indicated.


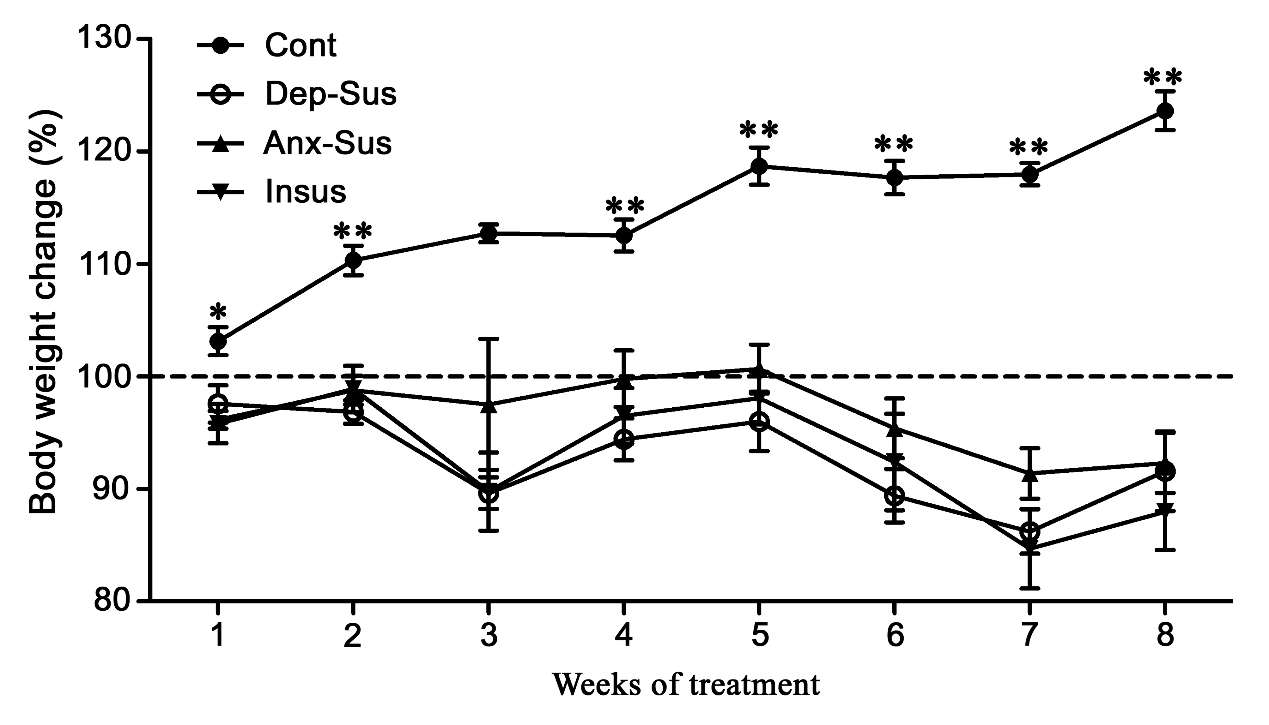


**Supplementary Figure S1**


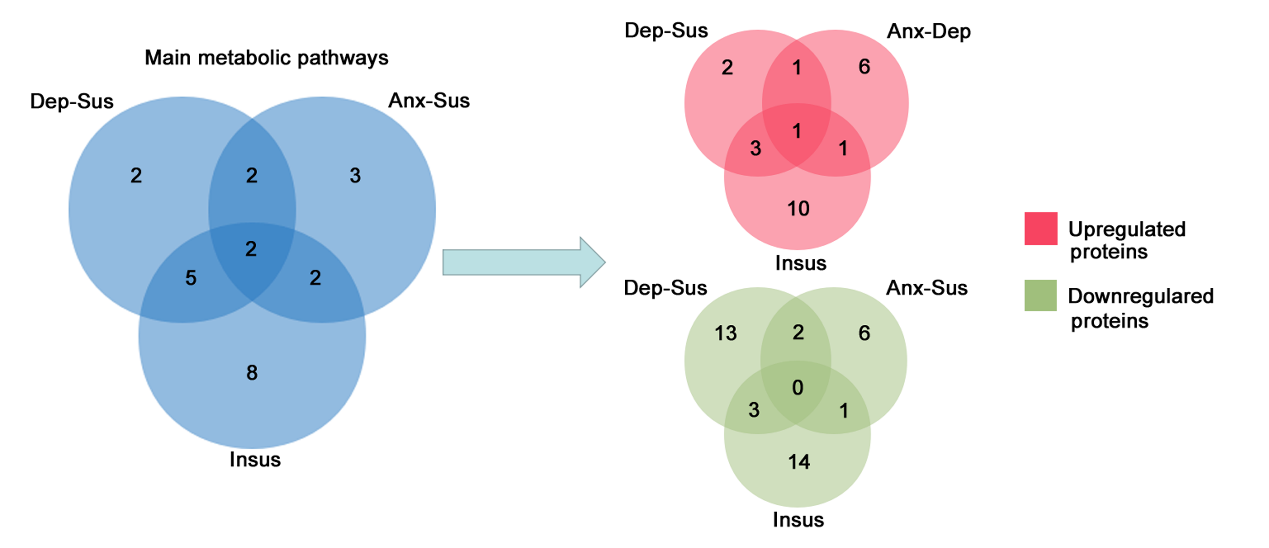


**Supplementary Figure S2**





**Supplementary Figure S3**

|  |  | **Sunday** | **Monday** |  | **Tuesday** |  | **Wednesday** | **Thursday** |  | **Friday** | **Saturday** |
| --- | --- | --- | --- | --- | --- | --- | --- | --- | --- | --- | --- |
| Paired housing | → | 8:00 |  |  | 20:00 | → | 12:00 |  |  |  | 17:00 |
| 45° cage tilt |  |  | 14:00-  20:00 |  |  |  |  |  |  |  |  |
| Soiled cage |  |  |  |  |  |  |  | 18:00 | → | 8:00 |  |
| White noise |  |  |  |  |  |  |  |  |  | 12:00-14:00 |  |
| Water deprivation |  |  |  |  | 20:00 | → | 7:00 |  |  |  |  |
| Empty water bottle |  |  | 8:00-  9:00 |  |  |  |  |  |  |  |  |
| Strobe light |  | 13:00-  16:00 |  |  |  |  | 19:00-  20:00 |  |  |  |  |
| Continuous lighting |  |  | 19:00 | → | 7:00 |  |  | 20:00 | → | 8:00 |  |

**Supplementary Table S1**
